# Data on mitochondrial function in skeletal muscle of old mice in response to different exercise intensity

**DOI:** 10.1016/j.dib.2016.04.043

**Published:** 2016-04-26

**Authors:** Chounghun Kang, Wonchung Lim

**Affiliations:** a Laboratory of Physiological Hygiene and Exercise Science, School of Kinesiology, University of Minnesota at Twin Cities, Minneapolis, MN 55455, USA; b Department of Sports Medicine, College of Health Science, Cheongju University, Cheongju 363-764, South Korea

**Keywords:** Mitochondria, Exercise, Skeletal muscle, Aging, Sarcopenia

## Abstract

Endurance exercise is securely linked to muscle metabolic adaptations including enhanced mitochondrial function (“Effects of exercise on mitochondrial oxygen uptake and respiratory enzyme activity in skeletal muscle” [1], “Effects of exercise on mitochondrial content and function in aging human skeletal muscle” [2]). However, the link between exercise intensity and mitochondrial function in aging muscle has not been fully investigated. In order to understand how strenuous exercise affects mitochondrial function in aged mice, male C57BL/6 mice at age 24 months were randomly assigned to 3 groups: non-exercise (NE), low-intensity (LE) and high-intensity treadmill exercise group (HE). Mitochondrial complex activity and respiration were measured to evaluate mitochondrial function in mouse skeletal muscle. The data described here are related to the research article entitled “Strenuous exercise induces mitochondrial damage in skeletal muscle of old mice” [3].

**Specifications Table**

**Table:** 

Subject area	*Biology*
More specific subject area	*Muscle biology*
Type of data	*Graph, table*
How data was acquired	*Mitochondrial respiration: Clark-type oxygen electrode with a mini-respiration chamber (Instech Laboratoryies, Inc.) Mitochondrial complex activity: Complex I–IV*
Data format	*Raw and analyzed data*
Experimental factors	*Mice were randomly assigned to 3 groups: non-exercise control group (NE), low-intensity exercise group (LE) and high-intensity exercise group (*HE*).*
Experimental features	*Mitochondria were isolated from mouse skeletal muscle*
Data source location	*Minneapolis, USA*
Data accessibility	*The data are included in this article.*

**Value of the data**

• This data demonstrate isolation of intact mitochondria in mouse skeletal muscle.
• This data first show a comparison of mitochondrial function in response to different exercise intensity in old mice.
• The data provide further insights into benefit of moderate-intensity exercise in aging muscle.

## 1 Data

**Fig. 1:**
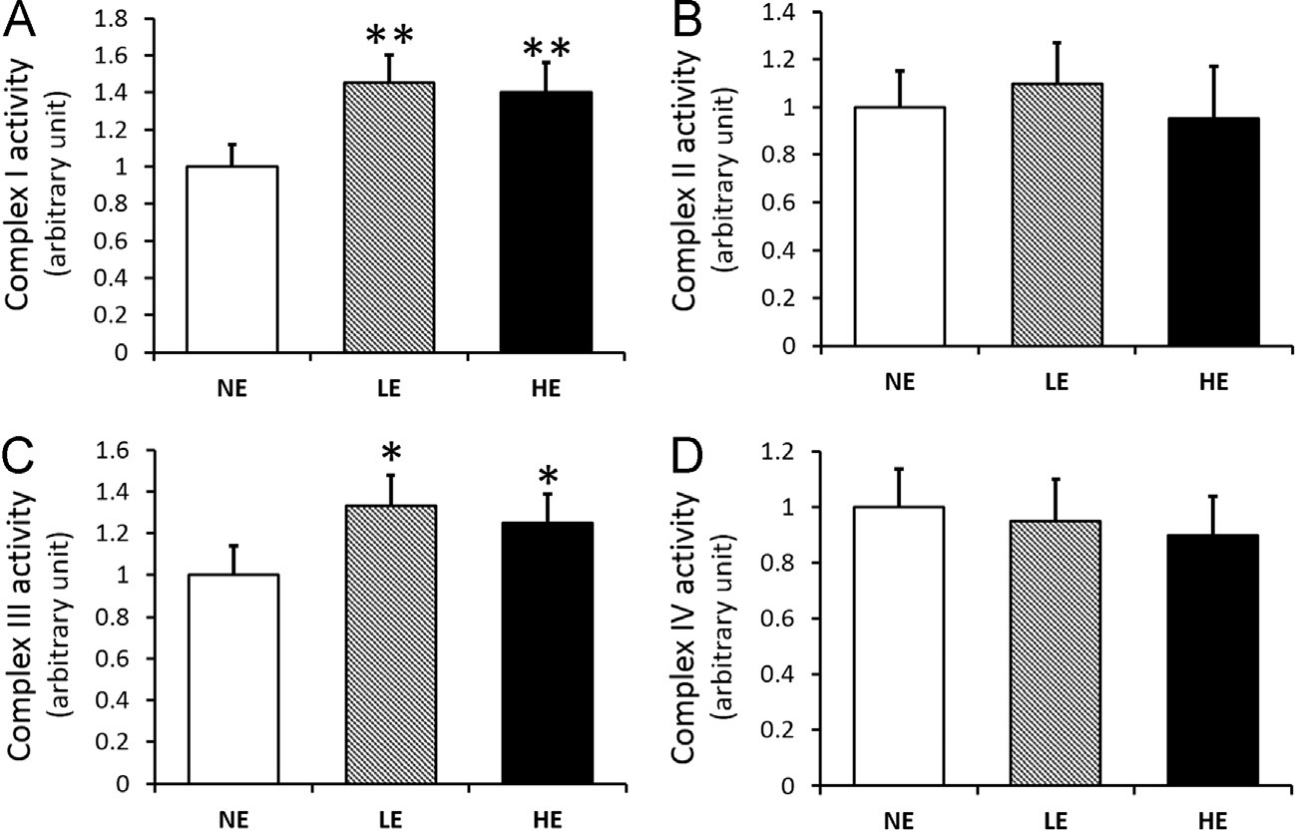
Effect of low- and high-intensity exercise on mitochondrial complex activity in old mouse soleus muscle: (A) the enzymatic activity of mitochondrial complex I (NADH: ubiquinone oxidoreductase), (B) complex II (succinate dehydrogenase), (C) complex III (decylubiquinol cytochrome c oxidoreductase) and (D) complex IV (cytochrome *c* oxidase) were measured in the isolated mitochondria. Values are the means±SEM; NE, non-exercise control group; LE, low-intensity exercise group; HE, high-intensity exercise group; *P<0.05 vs. NE; **P<0.01 vs. NE; one-way ANOVA with Tukey׳s HSD post hoc test.

**Fig. 2:**
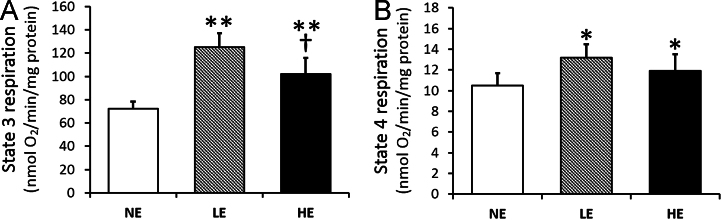
Effect of low- and high-intensity exercise on mitochondrial respiration in old mouse soleus muscle: (A) the state 3 and (B) state 4 respiration were measured in the isolated mitochondria. Values are the means±SEM; NE, non-exercise control group; LE, low-intensity exercise group; HE, high-intensity exercise group; *P<0.05 vs. NE; **P<0.01 vs. NE; ^†^*P*<0.05 HE vs. LE; One-way ANOVA with Tukey׳s HSD post hoc test.

The data presented here show the mitochondrial complex activity (Table 1 and Fig. 1), as well as mitochondrial state 3 & 4 respiration (Table 1 and Fig. 2) as an indicator of mitochondrial function.

## 2 Experimental design, materials and methods

### 2.1 Animals, experimental design

Male C57BL/6 mice at age 24 months were housed in temperature-controlled rooms (22 °C), on a reverse 12-h light/dark cycle. After a 1-week acclimation, mice were randomly assigned to three groups: non-exercise (NE, *N*=10), low intensity treadmill exercise (LE, *N*=10) or high intensity treadmill exercise group (HE, *N*=10) [3].

### 2.2 Treadmill exercise training

Animals from the exercise groups were subjected to 5 days of exercise regimen on a treadmill, while control animals were exposed to daily handling and spent the same time on a treadmill. Exercise intensity and duration were gradually increased during the first week of exercise training from 5 min at a speed of 4 m/min for aged mice to a regular regimen and then increasing the speed 1 m/min per minute until exhaustion. Starting from the second week, for 5 days at 50 min a day, mice of the LE and HE groups ran on a motor-driven treadmill at 35% and 70%, respectively, of the speed at which the mice reached exhaustion. Thus, the LE and HE were run at 8.8 and 17.45 m/min, respectively [3].

### 2.3 Mitochondrial isolation

Following the exercise session, each mice was sacrificed and soleus skeletal muscle tissues were quickly dissected. Mitochondria were isolated via differential centrifugation as previously described [4]. Briefly, 5 mL ice-cold 0.25 M sucrose, 1 mM EDTA, 5 mM HEPES, 0.2% bovine serum albumin (BSA), 13 units/10 mL collagenase (pH 7.4) isolation media 1 (IM1) was added and the muscle was minced with scissors. IM1 was then added to a 1:10 ratio (w/v) and the mixture was kept on ice for 30 min to allow for collagenase function. Using a Potter–Elvehjem homogenizer, the mixture was further homogenized with a maximum of five passes and the homogenate was filtered through 2 layers of sterile gauze, and spun at 4 °C for 10 min at 700*g*. The supernatant was saved on ice and the pellet was resuspended in a 1:10 ratio (w/v) of IM1 and spun again under the same conditions (4 °C for 10 min at 700*g*). The pellet was discarded and the combined supernatants were spun at 4 °C for 10 min at 12,000*g*. The resulting supernatant was discarded and fat on the centrifuge tube was removed with a sterile cotton-tipped applicator. The pellet was resuspended using a smooth-headed Potter–Elvehjem pestle in 15 mL of 0.25 M sucrose and 1 mM EGTA (pH 7.4) isolation media 2 (IM2). The mixture was again spun under the same conditions (4 °C for 10 min at 12,000*g*), the supernatant discarded, and fat removed. The pellet was resuspended in 200 μL of 0.25 M sucrose and 2 mM EDTA, pH 7.4 isolation media 3 (IM3) buffer using a smooth-headed Potter–Elvehjem pestle. The isolated mitochondria were kept on ice until use.

### 2.4 Mitochondrial complex activity

The enzymatic activity of mitochondrial complex I (NADH: ubiquinone oxidoreductase), complex II (succinate dehydrogenase), complex III (decylubiquinol cytochrome *c* oxidoreductase) and complex IV (cytochrome *c* oxidase) were measured in the isolated mitochondria as previously described with minor modifications (Fig. 1) [5], [6].

### 2.5 Mitochondrial respiration

Mitochondrial respiration was completed at 30 °C using a Clark-type oxygen electrode with a mini-respiration chamber (Instech Laboratories, Inc., system 600B, electrode model 125/05) with a YSI, Inc. (model 5300) signal conditioner and recorded with LabVIEW software. All measurements were completed using 2 mM pyruvate–malate as substrates in 130 mM KCl, 5 mM MgCl_2_, 20 mM NaH_2_PO_4_, 20 mM Tris, and 30 mM dextrose, pH 7.4 respiration media (RM) buffer. Following the addition of 20 μl (0.2–0.4 mg) mitochondria and substrate, 300 nmol of ADP was added to stimulate state III respiration. State IV respiration was measured with oligomycin (2 mg/l) in the absence of ADP phosphorylation (Fig. 2).

## Figures and Tables

**Table 1:** Mitochondrial complex activity and respiration following treadmill exercise.

	NE	LE	HE
Complex I activity	432±56.2	626.4±73.4⁎⁎	604.8±77.8⁎⁎
Complex II activity	36±3.60	39.6±3.96	34.2±3.97
Complex III activity	1352±202.8	1798±216.3⁎	1690±209.6⁎
Complex IV activity	522±88.74	495.9±99.18	469.8±93.96
State 3 respiration	72±60	125±10⁎⁎	102±12⁎⁎, †
State 4 respiration	10.5±1.3	13.2±1.4⁎	11.9±1.7⁎

All values are mean±SEM. Units: nmol/min/mg mitochondrial protein for complex I–IV activity; nmol O_2_/min/mg mitochondrial protein for mitochondrial State 3 & 4 respiration. Non-exercise group (NE, *n*=10); Low-intensity exercise group (LE, *n*=10); High-intensity exercise group (HE, *n*=10).

⁎⁎ *P*<0.01.

⁎ *P*<0.05 vs. NE.

† *P*<0.05 HE vs. LE.
